# Lower gastrointestinal bleeding due to colonic fistula caused by a gossypiboma: Case report and literature review

**DOI:** 10.1016/j.ijscr.2020.05.053

**Published:** 2020-05-29

**Authors:** María José Gómez-Jurado, Anna Curell, Rocío Martín, Amador García Ruiz de Gordejuela, Manel Armengol

**Affiliations:** a Department of General and Digestive Surgery, Hospital Universitari Vall d’Hebron, Barcelona, Spain; b Universitat Autonoma de Barcelona, Spain

**Keywords:** Gossypiboma, Retained surgical item, Foreing body, Lower gastrointestinal bleeding, Colonic fistula, Complication

## Abstract

**Introduction:**

Even in modern surgery, human mistakes cannot be totally avoided. Retained surgical items are among the most feared ones. Forgotten sponges inside patients can cause a wide range of complications due to the foreign body reaction, called gossypiboma. The incidence of gossypibomas in the literature is probably underreported due to its legal implications; however, we must know its consequences and highlight the importance of the prevention strategies. We present a case where only preventive measures would have avoided its fatal outcome.

**Presentation of case:**

An 85-year-old male, previous left nephrectomy 12 years before, came to the emergency room with hematochezia and hemodynamic instability. An emergent angio-CT revealed a 12 cm mass due to a gossypiboma near the descending colon; the presence of air suggested an infection and/or fistulization to the bowel. It was decided not to perform invasive procedures, resulting in the patient’s death.

**Discussion:**

Gossypibomas can remain asymptomatic for years, being diagnosed when causing an obstruction, malabsorption, septic symptoms or even spontaneously. This may lead to high morbidity and mortality rates. In order to prevent it, different strategies have been described, with the objective to intensify surveillance. When diagnosed, laparotomy, laparoscopic and even endoscopic procedures have been performed successfully.

**Conclusion:**

We suggest putting all our efforts in identifying high risk patients and surgeries, training the OR team and enhancing protocols and checklists to minimize any preventable errors.

## Highlights

• Retained foreign bodies may cause severe complications.
• Gossypiboma should be removed in all cases.
• Conservative treatment for retained foreign bodies may cause complications at any time.
• Prevention for retained foreign bodies is mandatory.

## 1 Introduction

Intraabdominal surgery can sometimes lead to human errors. One of them is leaving surgical items inside patients’ bodies. Specifically, forgetting a sponge inside a patient can cause a foreign body reaction surrounding the textile matrix [1,2]. This reaction is called “gossypiboma”, with an estimated incidence of 1/1000 to 1/1500 of intraabdominal operations. Real incidence of gossypibomas is probably underreported, mostly due to the legal implications of their detection, but also because many patients remain asymptomatic [2]. Such incidents may result in major injury. In a report of 24 cases of foreign bodies retained after intraabdominal surgery, complications observed included perforation of the bowel, sepsis, and death in two patients [1]. Transmural migration of an intra-abdominal gossypiboma has been reported to occur in the stomach, duodenum, ileum, colon, bladder, vagina and diaphragm [3].

We aim to present a case where only accurate prevention would have avoided its fatal ending. This paper is presented in line with the SCARE criteria [4].

## 2 Case presentation

An 85-year-old male, with history of left nephrectomy 12 years before due to a renal carcinoma, came to the emergency room (ER) by ambulance with hematochezia and hemodynamic instability. Patient’s comorbidities included hypertension, type 2 diabetes, chronic obstructive pulmonary disease and congestive heart failure. Patient was fully dependent for basic daily activities.

During the follow-up of his renal malignancy, a foreign body was identified at the first control CT scan. The patient was informed and declined a new surgery because of the high risk the new intervention would entail. A conservative management with radiological surveillance was decided.

**Fig. 1:**
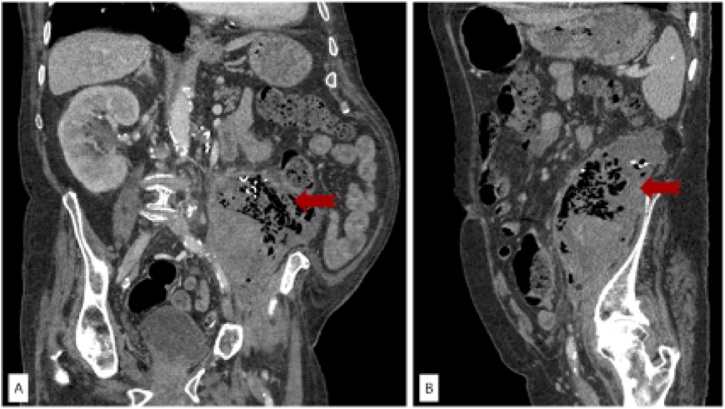
Lower gastrointestinal bleeding due to a colonic fistula caused by a gossypiboma A) Coronal view. B) Sagittal view.

The physical examination revealed left-side abdominal tenderness, associated to anemization, that required blood transfusion, and septic parameters in blood-test. An emergent angio-CT revealed a 12 cm mass due to a gossypiboma near the descending colon (Fig. 1). The presence of air inside the mass suggested an infection and/or fistulization to the bowel. No signs of active bleeding were observed. Due to the patient’s age and comorbidities, it was decided not to perform any aggressive procedures. The decision was discussed and accorded with the patient’s relatives because the patient was quite disorientated. We initiated full-dose wide-spread intravenous antibiotics and basic support measures. After 8 days of conservative treatment, the patient developed septic shock related to perforation of the sigmoid colon and pulmonary and heart failure, resulting in the patient’s death.

## 3 Discussion

The case we present represents a fatal and even unexpected evolution of an already known complication. The patient had been properly informed of the complication and non-surgical management was decided due to high comorbid condition of the patient. During evaluation of the patient at the ER, we considered that laparotomy and colectomy were the only surgical options for the patient. We found that no other less invasive approaches were eligible.

Several studies have tried to identify the risk factors of retained surgical items (RSI). All the following items are related to greater propensity for disorganization and team communication breakdowns, being more difficult to keep track of materials and having an increased likelihood of RSI [5]:

• Unplanned or unforeseen changes
• More than one subprocedure with or without multiples surgical teams
• Unplanned intraoperative events
• Multiple surgeons on various surgical teams
• Use of temporizing maneuvers, such as cavitary packing with surgical sponges, temporary vascular shunting, leaving *in situ* clamps and other surgical instruments during patient transfers
• Diverting team’s attention to items such as non-functioning equipment

On the other hand, Moffat-Bruce et al. showed in their meta-analysis that performing surgeries at dawn, emergency procedures and intraoperative changes in nursing staff, didn’t appear to have a significantly risk for RSI [5].

Obesity is a controversial item, being a significant risk factor in some studies [1,6], without reaching statistical significance in others [7]. Nevertheless, in all these studies patients with higher mean BMI were found in the RSI group, having similar anatomic locations for the RSI.

Lu et al. published two types of reaction secondary to gossypibomas [3]. The first one is an aseptic fibrous response to the foreign material that creates adhesions and encapsulation. This may take a silent clinical course which can be asymptomatic. A gossypiboma may undergo calcification, disruption, partial absorption, and even diffusion. In the abdomen, the sponge can get surrounded by omentum and intestines, which attempt to encapsulate it [8,9].

The second type of foreign body reaction is exudative, and produces an inflammatory reaction that ends with an abscess formation [3]. The body may attempt to extrude the foreign material, which can lead to complications such as erosion, external fistula formation and perforation of an adjacent viscera and migration. The small intestine is the most common viscera into which migration takes place. This type of response often causes symptoms like abdominal pain, nausea, vomiting, anorexia and weight loss, resulting from obstruction or a malabsorption-type syndrome caused by multiple intestinal fistulas or intraluminal bacterial overgrowth [3,8,9].

Some studies suggest that the gold standard should be a CT scan [8]. A characteristic sign of this pathology is the curved or banded radiopaque lines on plain X-ray or CT scan, which represent the radiopaque markers of the surgical swabs [8,10]. Nevertheless, this radiographic finding may not be seen in some cases, like in calcification depositions. Some surgical swabs used long time ago may not have radiopaque markers. We can find a rounded mass with a dense central part and an enhancing wall [[10], [11], [12]]. Gibbs et al. suggested that the spongiform pattern with entrapped gas bubbles, similar to what we found in our case (Fig. 1, is the most specific CT finding for gossypibomas, with an incidence of 54% [8].

Lu et al. described in their work a novel imaging feature called the “calcified reticulate rind”, consisting in a gradual deposition of calcifications along the fiber network of the surgical gauze [11]. Gossypibomas on MRI may appear as low-signal-intensity lesions on T2 weighed images with wavy, stripped or spotted appearances [8,10,12].

Gossypibomas should be removed as soon as diagnosed, being the gold standard method for treatment [3]. Different techniques, using laparoscopy and laparotomy, are used depending on the clinical presentation, localization and medical equipment available. According to the literature, gastrostomy and segmental resection, laparoscopic or laparotomy were used to remove gossypibomas that had migrated intraluminary. In those scenarios, endoscopic retrieval of foreign bodies has been reported [1] and may be indicated.

Gossypiboma-associated mortality is as high as 11–35% [13], stressing the importance of preventing this complication. However, every case must be evaluated individually, and a tailored balance should be considered [1,2].

Prevention of these potentially harmful events is critical to avoid the so called “never events”. The goal is to make that name come true. Training directed at increasing situational awareness and improved team communication should be in the first line of prevention. It would help to prepare operative teams to approach more effectively such unplanned events [5,6].

One of the most important strategies consists on enhancing careful and thorough swabs and material counting, and in cases of discrepancy, a thorough cavitary exploration and/or radiographic imaging of the surgical field must be performed [1,2,9,14,15]. Besides, there are some studies that encourage us to classify the patients into high or low risk. Using this tool would heighten awareness and understanding of the entire operating room, so to eliminate the occurrence of this unwanted events [1,5,14,15].

Gawande et al. showed that, among the many cases of retained foreign bodies in which counts were performed, 88% involved a final count that was erroneously thought to be correct [1]. In the same line, when radiographic imaging is performed, Stawicki et al. highlighted the fact that nearly half of all RSI were missed on initial interpretation of such “foreign body films”, giving the surgical team a false sense of security [6]. It is described that false-negative intraoperative radiographies (IOR) have resulted from poor quality films, multiple known radiopaque densities, and the radiologists being unaware of the concern about a foreign body [6,14].

More recently, different devices have been introduced to help detecting RSI using radiofrequency and other forms of electronic tagging [16]. The use of radiofrequency identification systems has a number of inherent disadvantages and has never come into general usage, probably because it is expensive, time consuming and involves radiation, albeit a minimal amount. Moreover, other foreign bodies that are nonmetallic and do not contain a radiopaque marking will not be imaged on a radiograph.

The results of the testing described by Fabian et al. suggest that electronic technology could reduce the incidence of retained items [16]. This system would have the added advantages of being portable and quick, not using radiation, and discovering retained sponges while the wound is still open. However, since no electronic device can be completely secure and foolproof, such a system could not replace the traditional means of inventory and search, but would instead be used in conjunction with it.

Routine IOR is the most cost-effective strategy in preventing RSI [14]. However, the main conclusion is that you spend as much as you save. Furthermore, the incidence rate of RSI is low, so it can take a long time to realize the true cost savings that these measures would entail.

## 4 Conclusion

This case report showed us the importance to put all our efforts in preventive measures. After knowing the fatal evolution, the original conservative management can be thoroughly discussed. It is essential to identify high risk patients, train the OR team, insist in an accurate counting and consider other emerging forms of electronic tagging. Gossypibomas are difficult to be identified in the early post-operative due to their silent course and, at the time of the initial symptoms, their removal may be associated with a high complication and mortality rate. Overall, prevention is the key to turn this condition into a real “never event”.

## Author contribution

**María José Gómez-Jurado:** Managed the case, Writed the original manuscript, Got informed consent.

**Anna Curell:** Managed the case, Reviewed and edited the manuscript, Got informed consent.

**Rocío Martín:** Managed the case, Reviewed and edited the manuscript, Got informed consent.

**Amador García Ruiz-de-Gordejuela:** Managed the case, Reviewed and edited the manuscript.

**Manel Armengol Carrasco:** Reviewed and edited the manuscript, Supervised the process.

## Funding

This research did not receive any specific grant from funding agencies in the public, commercial, or not-for-profit sectors.

## Ethical approval

The study was exempted from ethical approval.

## Informed consent

Relatives were contacted and signed informed consent for the publication.

## Registration of research studies

NA.

## Guarantor

Amador García Ruiz de Gordejuela.

## Provenance and peer review

Not commissioned, externally peer-reviewed.

## Declaration of Competing Interest

The authors declare no conflict of interest.
